# Biochar Utilization in Antimicrobial, Anticancer, and Biosensing Applications: A Review

**DOI:** 10.3390/biom15060760

**Published:** 2025-05-25

**Authors:** Ki Ha Min, Koung Hee Kim, Joo-Hyung Seo, Seung Pil Pack

**Affiliations:** 1 Institute of Industrial Technology, Korea University, Sejong 30019, Republic of Korea; alsrlgk@gmail.com; 2 Department of Biotechnology and Bioinformatics, Korea University, Sejong 30019, Republic of Korea; wood1018@korea.ac.kr (K.H.K.); sjh0413@korea.ac.kr (J.-H.S.)

**Keywords:** biochar, biomedical application, antibiotics, anticancer, biosensor

## Abstract

Biochar, a carbonaceous material derived from biomass, has garnered significant attention for its biomedical applications due to its unique physicochemical properties. Recent advances in functionalized and composite biochar materials have enabled their use in antibacterial and anticancer treatments, as well as biosensing technologies. This review highlights recent advances in the use of biochar for antimicrobial, anticancer, and biosensing applications. Derived from plant-, marine-, or animal-based biomass through pyrolysis, biochar can be functionalized with silver nanoparticles, metal oxides, or polymers to enhance its antimicrobial activity. In anticancer research, biochar demonstrates the ability to inhibit cancer cell proliferation, modulate the cell cycle, and deliver targeted therapeutics, showing selective cytotoxicity against specific cancer cell types. Furthermore, biochar-based biosensors, when integrated with biomolecules such as enzymes, DNA, or antibodies, exhibit high sensitivity and specificity, making them suitable for precise disease diagnostics. These findings suggest that biochar holds significant potential as a sustainable biomedical material, offering alternatives to conventional antibiotics, supporting cancer therapy, and enabling sensitive biosensing platforms. Future functionalization strategies may further facilitate its clinical translation and practical applications.

## 1. Introduction

Biochar is a carbon-rich, porous material produced through the pyrolysis of biomass or other organic materials under oxygen-deficient conditions, typically at temperatures ranging from 300 to 900 °C [1,2,3,4]. There are several sources for it, including agricultural residues (for example, wheat straw, rice husk, and corn), forestry waste, municipal waste (for example, dewatered sludge), aquatic biomass, and industrial wastes [5,6,7,8]. As a product of biomass feedstock utilization, biochar aligns with the principles of green chemistry and has gained increasing attention for its diverse applications [9,10,11,12].

**Figure 1:**
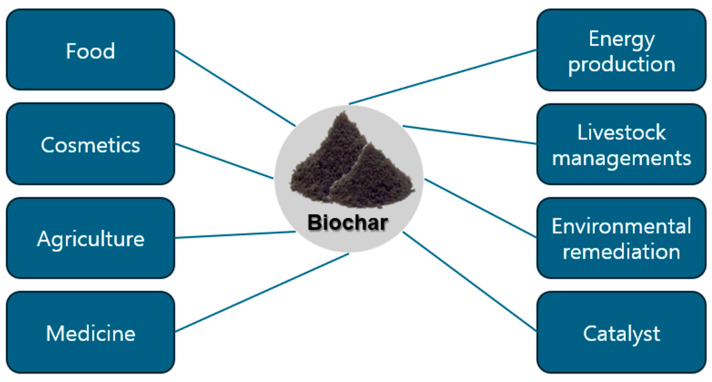
Overview of potential applications of biochar across various fields. Biochar has diverse applications, including its use in food, cosmetics, agriculture, and medicine. Additionally, it plays a role in energy production, livestock management, environmental remediation, and catalysis, highlighting its multifunctional benefits in industrial and ecological sectors.

In addition to carbon (C), biochar contains ash, hydrogen (H), oxygen (O), nitrogen (N), and sulfur (S), along with various functional groups such as phenolics, alcohols, and hydroxyl aromatics [13,14,15]. These characteristics contribute to its alkaline pH, high thermal and chemical stability, well-developed porosity, and large specific surface area, making it valuable in numerous applications [10,11,12]. Its exceptional adsorption capabilities stem from its extensive surface area and abundant active sites [16]. Furthermore, biochar can act as a catalyst in various reactions through the generation of persistent free radicals (PFRs) and can serve as a carrier for functional chemicals [17]. Biochar properties can be further enhanced through modification techniques, including physical treatments (e.g., optimizing size and density), chemical activation (e.g., acid, metal salt, and alkaline modifications), and biological engineering approaches (e.g., microorganism colonization and doping with other materials) [18,19,20]. These attributes, along with its stability, environmental sustainability, and ease of production, make biochar a promising material for industrial, environmental, and biomedical applications [21,22,23,24] (Figure 1).

The increasing costs of healthcare, combined with the growing global population and rising prevalence of chronic diseases, highlight the need for cost-effective and multifunctional biomaterials with high precision and minimal adverse effects [23,24,25]. Conventional biomaterials, including metals, ceramics, and synthetic polymers, often present challenges related to biocompatibility, biodegradability, and functional limitations [21,22,26]. Consequently, there is growing interest in alternative materials with enhanced properties for biomedical applications [27,28,29].

**Figure 2:**
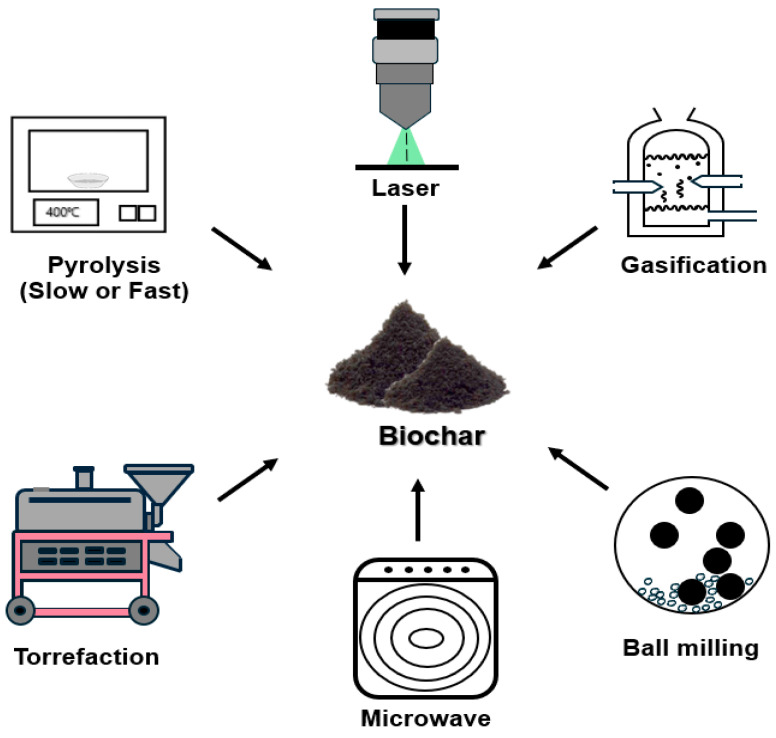
Schematic representation of biochar production and modification processes.

Biochar, magnetic biochar, and other biomass-derived carbonaceous materials have recently gained attention for their potential biomedical applications [26,30]. These materials offer several advantages, including high surface area, tunable porosity, chemical stability, and functional groups that facilitate biointeractions [31,32,33,34]. Their biocompatibility and modifiability make them promising candidates for applications such as drug delivery, tissue engineering, biosensing, and regenerative medicine [27,28,29]. Additionally, biochar-based materials can be produced from renewable biomass sources, making them a cost-effective and environmentally sustainable alternative to synthetic nanomaterials [25,35,36,37] (Figure 2).

Biochar has garnered significant attention across various fields, including wastewater treatment, energy storage (capacitors), sensors, and environmental management, due to its unique chemical properties, cost-effectiveness, abundant availability, and environmental sustainability [37,38,39,40]. With the growing global emphasis on sustainable development, researchers are actively exploring eco-friendly alternatives by improving raw materials, optimizing manufacturing processes, and utilizing byproducts more efficiently [41,42,43].

Recently, the biomedical applications of biochar have emerged as a promising area of study, demonstrating potential in fields such as drug delivery, tissue engineering, and antimicrobial treatments [23,44]. However, despite its promising characteristics, a comprehensive review detailing the biomedical relevance of biochar remains lacking. This paper aims to systematically assess the biomedical potential of biochar, focusing on its biocompatibility, functional properties, and safety considerations. Additionally, it critically examines the key challenges and limitations that must be addressed for the successful clinical translation of biochar-based materials. By identifying current research gaps and proposing future research directions, this review seeks to provide valuable insights into advancing biochar applications within biomedicine.

## 2. Preparation and Functionalization of Biochar

Biochar is a carbonaceous material derived from the thermal and mechanical processing of biomass, which plays a significant role in environmental remediation, carbon sequestration, and various industrial applications. The primary methods for biochar production include pyrolysis, gasification, torrefaction, and ball milling, each of which influences its physicochemical characteristics. These methods influence the physicochemical properties of biochar, making it suitable for diverse applications [21,22,32,45].

### 2.1. Biochar Production

Pyrolysis is the most common biochar production method, involving the thermal decomposition of biomass in an oxygen-limited environment. It can be classified into slow pyrolysis, which occurs at lower temperatures (typically 300–500 °C) with longer residence times, leading to higher biochar yields, and fast pyrolysis, which operates at higher temperatures (typically 500–700 °C) with shorter residence times, favoring bio-oil and gas production while generating a lower biochar yield [41,46].

Gasification is a thermochemical conversion process conducted at higher temperatures (700–1000 °C) with a controlled supply of oxygen or steam, primarily producing syngas (a mixture of CO, H_2_, and CH_4_) while leaving behind a small amount of high-porosity biochar. This method is useful for energy applications but results in lower biochar yields compared to pyrolysis [41,47].

Torrefaction is a mild thermal treatment carried out at temperatures between 200 and 300 °C under an inert or low-oxygen atmosphere. This process removes moisture and volatile components from biomass, leading to increased energy density and improved grindability of the resulting biochar. Torrefied biochar retains more of the original biomass structure while exhibiting enhanced hydrophobicity and fuel properties [48,49,50].

Ball milling is a post-treatment modification technique that applies mechanical force to reduce the particle size of biochar. This process increases its surface area, pore structure, and reactivity, making it more effective for applications such as adsorption, catalysis, and soil amendment. Ball milling also promotes structural defects and enhances the functional groups on biochar, which can improve its performance in environmental remediation and energy storage applications [51,52].

The carbonization process plays a crucial role in biochar production, along with the selection of appropriate precursors [42]. While the choice of biomass is important, the selection of the carbonization method is equally significant. The chosen carbonization technique directly affects the chemical and physical properties of biochar, including its structural characteristics, surface area, porosity, chemical composition, functional groups, and degree of graphitization [53,54,55] (Table 1). Understanding these different biochar production and modification techniques is essential for tailoring its properties to meet specific needs in sustainable agriculture, pollutant removal, and carbon capture technologies [37,56,57].

### 2.2. Biochar Modification

**Figure 3:**
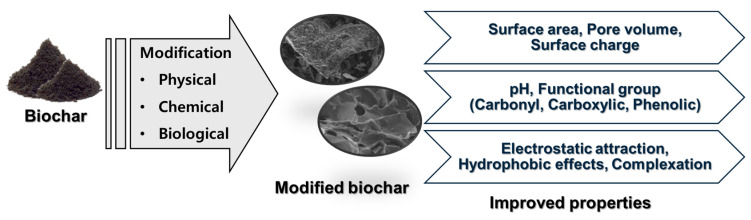
Schematic representation of biochar modification and its impact on physicochemical properties. Biochar undergoes various modification processes, including physical, chemical, and biological treatments, which enhance its structural and surface characteristics.

Different chemical modifications can significantly enhance the functionality and adsorption efficiency of biochar, including acid, base, and salt treatments, as well as other advanced modification techniques [89] (Figure 3). These treatments alter the physicochemical properties of biochar by introducing or enhancing functional groups (e.g., –COOH, –OH), increasing porosity, and improving surface charge characteristics. Through these modifications, biochar’s surface properties, porosity, and functional groups are improved, optimizing its adsorption capacity for pollutants [89].

Acid treatments, such as those hydrochloric acid (HCl), sulfuric acid (H_2_SO_4_), phosphoric acid (H_3_PO_4_), and nitric acid (HNO_3_), are commonly used for biochar modification [90]. Acid treatments improve textural parameters by removing impurities from the surface and developing micropores, thereby increasing biochar’s adsorption potential. Additionally, acid treatment enriches the biochar surface with oxygen-containing functional groups, particularly carboxyl groups [89]. For example, treatment with phosphoric acid enhanced the pore structure and increased the specific surface area of the biochar, thereby significantly improving its adsorption capacity. The modified biochar contained various functional groups, including hydroxyl, carboxyl, and carbonyl groups, which promoted hydrogen bonding and π–π interactions with enrofloxacin, thus strengthening hydrophobic interactions [91]. Treatment with alkalis such as potassium hydroxide (KOH) and ammonium hydroxide (NH_4_OH) is another common approach to modifying biochar. Alkali treatment increases the Brunauer–Emmett–Teller (BET) surface area, improves porosity, and enhances the concentration of oxygenated functional groups, which are crucial for adsorption [89].

Biochar modification with salts, such as chlorides and phosphates, can significantly alter its textural parameters and surface chemistry [89,92]. For example, pre-treatment with potassium phosphate (K_3_PO_4_) has been observed to enhance the adsorption capacity of biochar by modifying its surface structure. Additionally, iron-modified biochar has been demonstrated to be effective in mitigating the health effects of aqueous Cr(VI) contamination [93,94]. Hydrogen peroxide (H_2_O_2_) is an alternative modifying agent that is both cost-effective and environmentally safer compared to strong acids and bases [89]. H_2_O_2_ modification enhances biochar’s surface oxygen content and adsorption efficiency. For example, biochar derived from rape stalks and treated with H_2_O_2_ has demonstrated improved sorption of tetracycline [95,96]. Beyond these conventional modifications, biochar properties can be further enhanced through additional chemical processes. Techniques such as alkali, acid, and mineral reforming, as well as coating and oxygen plasma activation, employ chemicals like carbon disulfide, sodium hydroxide, and sulfuric acid to improve biochar’s adsorption potential [19,97].

In addition to inorganic modifications, organic substances such as polymers, organic acids, and vitamins can be applied to alter biochar properties [98,99,100]. Polymers, both synthetic (e.g., hydrogels) and natural (e.g., chitosan), play a crucial role in biochar modification by introducing new functional groups, particularly nitrogen-containing ones [101,102,103,104]. Both synthetic macromolecules, such as hydrogels, and natural compounds, such as chitosan, have been used to enhance biochar stability and adsorption performance. Polymer incorporation not only improves dimensional stability and hydrophobicity but also increases the atomic O/C ratio, as observed in chitosan-modified biochar. Such modifications significantly enhance biochar’s capacity to remove heavy metals from contaminated environments [105]. Citric acid-modified biochar has been shown to improve phosphorus availability in calcareous sandy soil, while vitamin B6-upgraded biochar exhibits enhanced adsorption of tetracycline from aqueous solutions [98]. Organic acid treatments, such as those involving citric and acetic acid, modify biochar’s surface chemistry by promoting functional group formation via esterification, although they may reduce porosity. In biochar prepared at high temperatures, organic acids can react with ash components, facilitating pore formation and altering structural properties [105]. This incorporation enhances biochar’s dimensional stability and hydrophobicity, further improving its performance in environmental applications [106,107,108].

Biological modification, particularly the use of microorganisms for heavy metal adsorption from aqueous solutions, has emerged as a cost-effective, efficient, and easily operable approach, especially for treating low-concentration heavy metal wastewater [22,109,110,111]. Residual biomass derived from industrial microorganisms, including bacteria, algae, fungi, and yeast, has demonstrated significant potential for heavy metal accumulation through various adsorption mechanisms [109,112]. The biosorption process in microorganisms involves multiple mechanisms, such as complexation, physical adsorption, cross-membrane transport, ion exchange, and precipitation, all of which contribute to the effective removal of heavy metals from contaminated water sources [113,114].

## 3. Biochar for Biomedical Applications

**Figure 4:**
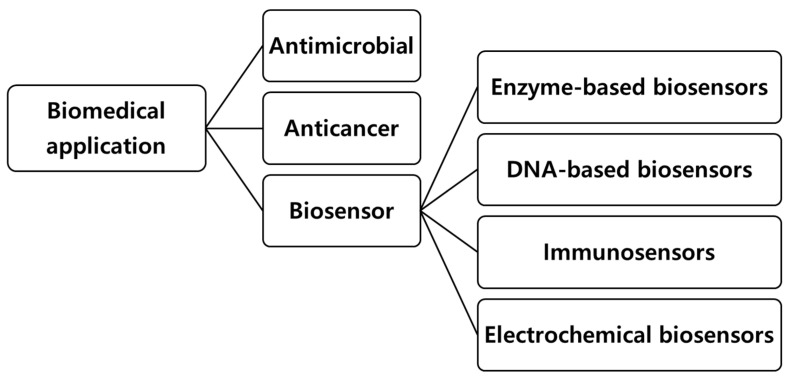
Schematic representation of biomedical applications of biochar and its role. Biochar demonstrates potential applications in the biomedical field, including antimicrobial and anticancer treatments. Additionally, biochar-based biosensors are categorized into enzyme-based biosensors, DNA-based biosensors, and immunosensors, which contribute to advanced diagnostic and therapeutic technologies.

Recent advancements in biomedical engineering have increasingly explored biochar as a multifunctional material with diverse therapeutic and diagnostic applications [21,22,25,26]. Derived from biomass through carbonization, biochar possesses unique physicochemical properties, such as a high surface area, tunable porosity, and rich functional groups, making it an excellent candidate for biomedical use [31,32,33,34] (Figure 4). Functionalized and composite biochar materials have demonstrated significant potential in antibiotic therapies, anticancer treatments, and biosensor development, addressing key challenges in drug resistance, targeted cancer therapy, and disease detection [27,28,29].

### 3.1. Biochar in Antimicrobial Applications

**Figure 5:**
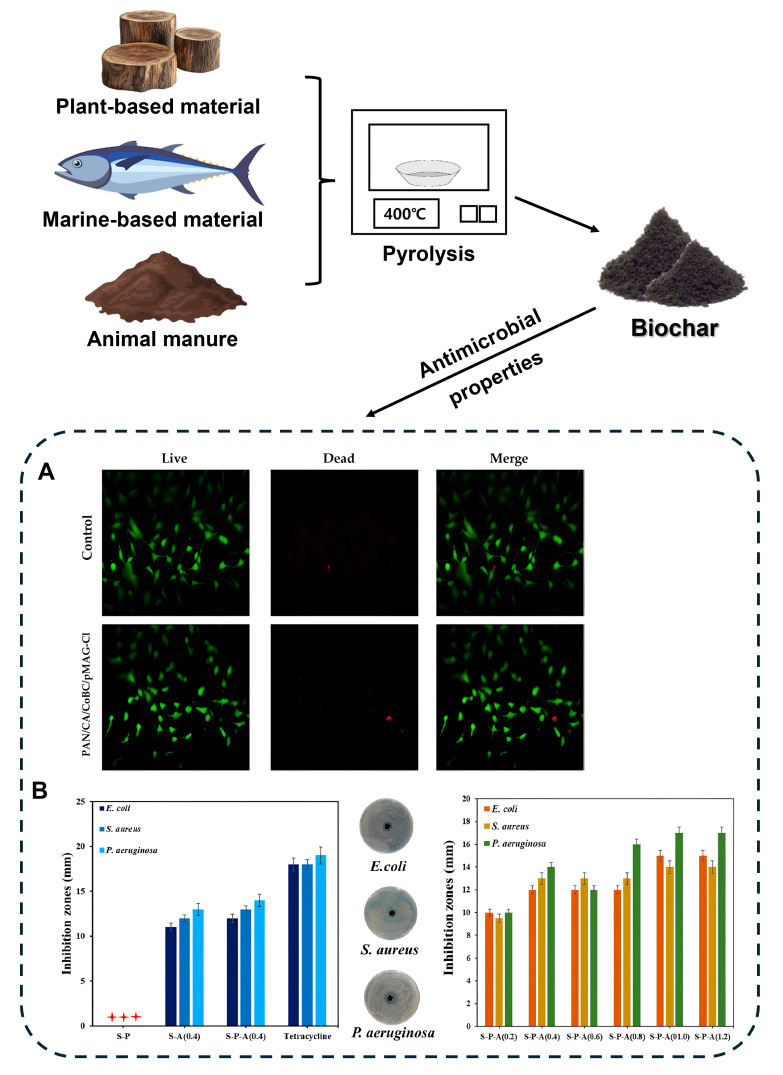
Schematic illustration of biochar production from various biomass sources and its antimicrobial mechanisms. Biochar can be produced via pyrolysis at 400 °C using different types of biomass including plant-based materials (e.g., wood), marine-based materials (e.g., fish), and animal manure. The resulting biochar can be tested for cell affinity and antimicrobial activity. (**A**) Reprinted with permission from [119], Copyright © 2024, American Chemical Society. (**B**) Reprinted with permission from Ref. [120]. “+++ ” means no antibacterial effect.

Biochar has gained significant attention as a multifunctional material with remarkable antimicrobial properties (Figure 5). Its high surface area, abundance of functional groups, and excellent adsorption capacity contribute to its antimicrobial activity. The porous surface with functional moieties can interact with bacteria, leading to cell adhesion and membrane disruption, thereby preventing biofilm formation. Additionally, the generation of ROS by biochar is one of the reasons why biochar exhibits bacterial growth inhibition and killing effects. Various modification methods can enhance the mentioned effects and can also enhance upgraded antimicrobial effects such as sustained release systems or targeted delivery by combining with antimicrobial agents such as antibiotics, metal ions, enzymes, and peptides [115,116,117,118]. Numerous studies have demonstrated the potential of biochar functionalized with antimicrobial compounds to effectively suppress bacterial and fungal pathogens (Table 2).

Among the many biochar sources, plant-based materials have shown significant antimicrobial activity. Biochar derived from wood powder, rice husks [121], maize straw [122], potato peels [123], coconut husks [124], etc., have proven to be particularly effective in controlling microbial growth. The addition of metal ions or organic antimicrobial agents to these plant-based biochars improves their ability to disrupt bacterial membranes and inhibit microbial proliferation [122]. Notably, maize straw biochar, when functionalized with iron oxide or phosphonium compounds, has been demonstrated to achieve nearly 100% bacterial removal, even at high concentrations of 10^6^ CFU/mL with a dosage as low as 20 mg/L.

Recently, considerable research has been conducted on biochar derived from marine sources. Biochar from dried fish scales [120] and waste fish scales [125,126] is particularly effective due to its high mineral content, which enhances its interaction with antimicrobial agents such as silver and chitosan [125]. Studies have shown that fish scale-derived biochar, modified with silver nanoparticles or nanocellulose, can effectively inhibit bacterial cell wall formation, disrupt protein synthesis, and interfere with ion transport channels. Inhibition zones exceeding 20 mm have been observed against Pseudomonas aeruginosa, Staphylococcus aureus, and Escherichia coli when the chitosan dosage reached 15 g/L.

Further expanding the range of biochar sources, those derived from animal byproducts such as cow dung [119], hazelnut shells [127], and barley distillers’ grains [128] have also demonstrated significant antimicrobial activity. This biochar, when functionalized with metal-based or polymeric agents, has shown great potential in applications such as biosensing, drug delivery, and wound healing [119]. In particular, cow dung-derived biochar, when incorporated into an N-halamine hydrogel, has proven effective as an antimicrobial wound dressing, aiding in infection prevention and promoting tissue regeneration. Wound healing assessments revealed that, by the seventh day of treatment, wounds treated with the cow dung biochar-modified hydrogel exhibited substantial granulation tissue formation and complete epidermal regeneration, a notable improvement compared to the control group.

When combined with antimicrobial agents, biochar demonstrates strong bactericidal and fungicidal effects through its various mechanisms. By disrupting cell membranes, interfering with protein synthesis, and inhibiting nutrient uptake, biochar emerges as a promising material for a range of applications, including wound healing [119], biosensing, drug delivery [127], and infection control [124]. Future research should focus on optimizing biochar modifications to enhance its stability, efficacy, and biocompatibility for broader biomedical applications.

**Table 2:** Antimicrobial applications of modified biochar.

Source	Additional Material	Methods	Target	Effects	Ref.
Cow dung	Photothermal N-halamine hydrogel	Pyrolysis acidification	*E*. *coli*, *S*. *aureus*	- Promotes the release of free halogen due to binding biochar - Thermal stability maintained 78.4 °C after 5 NIR cycles - 20–30% increased release of active chlorine as bactericidal agent	[119]
Wood powder and rice husk	Commiphora myrrha (T. Nees)	Oven-dried at 100 °C for 24 h. Pyrolysis (550 °C, 2 h)	*S*. *aureus*, *P*. *aeruginosa*, *S*. *Enteritidis*	- Improving material stability by adsorption onto solid biochar	[121]
Hazelnut shells	Boric acid (H_3_BO_3_) solutions	Pyrolysis (500 °C, 10 °C/min, 1 h)	*C*. *albicans*, *S*. *aureus*	- Reduces the formation of PAHs by up to 85% when combined with biochar - Doped with boron inhibit mycelial growth and the growth of *Candida albicans* yeasts	[127]
Maize straw	Iron oxide, quaternary phosphonium salt	Pyrolysis (500 °C, anoxic condition)	*E*. *coli*, *S*. *aureus*	- Effective penetration of bacterial cell membranes due to biochar - Enhanced the biocide effect to penetrate the cell wall and membrane into the cytoplasm	[122]
Waste barley distillers’ grains shell	Silver nanoparticles, polyvinyl alcohol-chitosan	Pyrolysis (300 °C, 2 h)	*E*. *coli*, *S*. *aureus*	- Prevents aggregation of silver nanoparticles by immobilizing them in biochar - Spike in protein/nucleic acid leakage in *E. coli* and *S. aureus* within 2 h	[128]
Potato peels	Glutaraldehyde, sodium alginate	Microwave pyrolysis (20 min, maximum microwave power)	*S*. *aureus*, *P*. *aeruginosa* *E*. *faecium*, *E*. *faecalis* *L*. *monocytogenes*	- Delivery vehicle for antimicrobial agents - Impair cell membrane permeability - Up to 84% antimicrobial effectiveness against strains used in experiments	[123]
Coconut husk	Polybutylene adipate terephthalate/Carvacrol	Pyrolysis (70 °C, 4 h and 80 °C, 12 h/vacuum-dried)	*E*. *coli*, *L*. *monocytogenes*, *S*. *enteritidis*, *S*. *aureus*	- Move through the peptidoglycan layer and act on the cytoplasmic membrane - Exhibits antimicrobial effects by spreading and disrupting the external lipopolysaccharide covering	[124]
Rice husk and cotton	Silver ion	Pyrolysis (480 °C, 5 °C/min, 3 h)	*E*. *coli*	- Adsorption of silver ions due to the high structural properties of biochar - Exhibited significant antibacterial properties (reducing *E. coli* colonies by 83% within 30 min)	[117]
*Atriplex halimus* L.	Ag–Cu	Pyrolysis (550 °C, 3 h)	*E*. *coli*, *K*. *pneumonia*, *B*. *subtilis* *S*. *aureus*	- Removal efficiency of 55.21% at the highest concentration (50 μg/mL) - Prevent bacterial DNA condensation and replication by causing hyperthermia and preventing nutrient uptake	[129]
Dried fish scale	Silver, polyvinyl alcohol, alginate gel beads	Pyrolysis (300 °C, 10 °C/min, 2 h)	*E*. *coli*, *S*. *aureus*, *P*. *aeruginosa*	- Synthesized with biochar to form a hydrophobic structure to improve silver ion release - At a gel bead dose of 0.50 g/L, the bacteriostatic effect was over 90%	[120]
Waste fish scale	Nanocellulose, silver, chitosan-polyvinyl alcohol hydrogel	Pyrolysis (300 °C, 2 h)	*E*. *coli*, *S*. *aureus*, *P*. *aeruginosa*	- Promotes the release of silver ions and amino groups to enhance antimicrobial effects - Above 95% antimicrobial within 10 days	[125]
Waste fish scale	Carbon substrate, nanosilver	Pyrolysis (500 °C, 10 °C/min, 2 h)	*E*. *coli*, *S*. *aureus*, *P*. *aeruginosa*	- Porous structure of biochar effectively loads silver ions - At 0.2 g/L dosage, antibacterial effect observed within 1 h	[126]

*E. coli: Escherichia coli; S. aureus: Staphylococcus aureus; P. aeruginosa: Pseudomonas aeruginosa; E. faecium: Enterococcus faecium; E. faecalis: Enterococcus faecalis; L. monocytogenes: Listeria monocytogenes; S. enteritidis: Salmonella enteritidis; K. pneumonia: Klebsiella pneumoniae; B. subtilis: Bacillus subtilis.*

### 3.2. Biochar in Anticancer Applications

**Figure 6:**
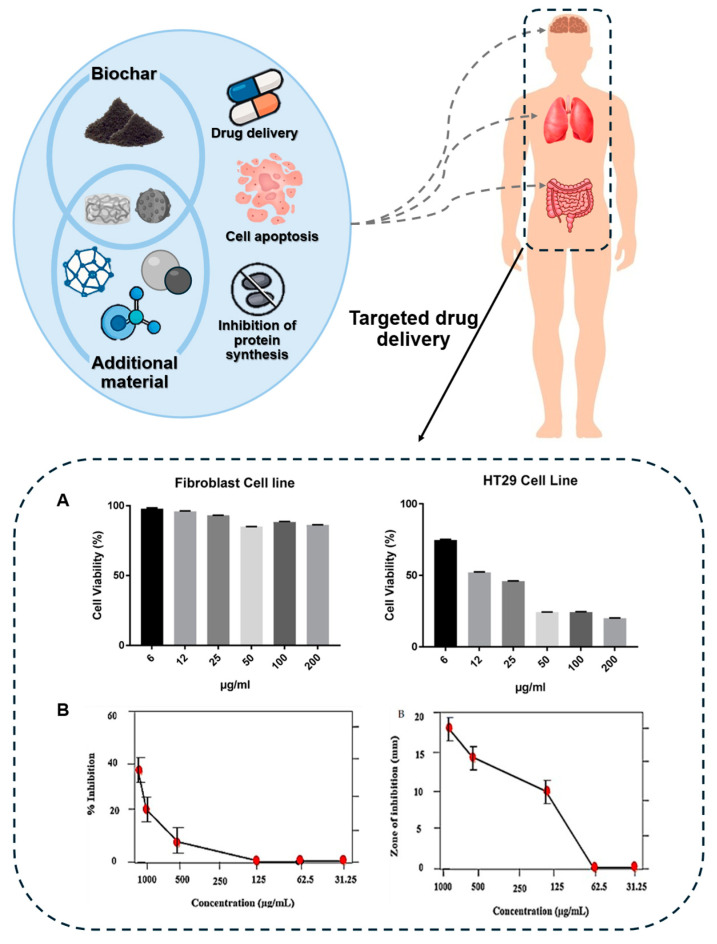
Potential anticancer applications of biochar and its composites. Biochar, alone or in combination with additional nanomaterials, has shown promise in cancer therapy through multiple mechanisms. These include targeted drug delivery, induction of cancer cell apoptosis, and inhibition of protein synthesis. (**A**) Reprinted with permission from Ref. [135]. (**B**) Reprinted with permission from Ref. [136].

Biochar, a carbon-rich material produced through the pyrolysis of organic biomass, has emerged as a promising candidate in the field of cancer therapy due to its unique physicochemical properties (Figure 6). Recently, it has been reported that the alkaline nature of biochar inhibits tumor growth by interfering with the creation of a weakly acidic microenvironment by acetic acid production in tumors, thereby exerting anticancer effects [130]. In addition, nanobiochar can penetrate cancer cells and generate ROS, which is expected to have an anticancer effect [131,132]. The adsorption and release control ability of biochar plays an important role in the specific delivery of functional substances and contributes to reducing the toxicity of drugs or nanoparticles that can damage normal cells due to their strong inhibitory effects [133,134]. Among the various investigations into the anticancer properties of biochar (Table 3), three studies stand out due to their distinctive approaches and significant findings.

One of the most noteworthy studies focuses on biochar derived from alder wood chips combined with butyrate glycerides [137]. This modified biochar demonstrated enhanced sensitivity in HCT116 colorectal cancer cells, suggesting that biochar can synergize with bioactive compounds to exert a 22% enhanced anticancer effect compared to the control. The incorporation of butyrate glycerides is particularly interesting as it may promote cell cycle arrest and apoptosis, mechanisms critical to cancer cell elimination. This research emphasizes the potential of biochar as an adjunct to precision medicine, where its ability to enhance the therapeutic efficacy of anticancer agents could significantly improve treatment outcomes for colorectal cancer.

Similarly, a recent study focused on orange peel-derived biochar processed via hydrothermal carbonization was found to form carbon nanostructures that function as effective drug delivery carriers [138]. When tested on A549 lung cancer cells, the carbon nanostructures improved the bioavailability of anticancer drugs, allowing for targeted delivery to the tumor site. Compared to DHF (5,5-dimethyl-6a-phenyl-3-(trimethylsilyl)-6,6a-dihydrofuro[3,2-b] furan-2(5H)-one) alone, treatment with the drug at a concentration of 10 µg/mL−1 resulted in a higher cell death rate, measuring 17.62% (±1.9) versus 9.63% (±1.1) for DHF alone. This finding highlights the potential of this approach in overcoming a key challenge in cancer therapy developing drug delivery systems that not only enhance therapeutic efficacy but also reduce systemic toxicity. This approach addresses a critical challenge in cancer therapy: the need for drug delivery systems that can minimize systemic toxicity while enhancing therapeutic effectiveness. The ability of orange peel-derived biochar to facilitate controlled drug release holds significant promise for improving the outcomes of lung cancer treatments.

A further distinct approach is seen in the study of biochar from Mangifera indica bark, which was enhanced with Ag/Cu-ZrO_2_ nanostructures [135]. This biochar demonstrated potent anticancer activity against the SH-SY5Y neuroblastoma cell line, with a concentration-dependent cytotoxic effect. At 200 µg/mL, the nanocomposite induced 73.61% cancer cell death, exhibiting inhibitory effects comparable to paclitaxel. The IC_50_ value was determined to be 33.61 ± 1.07 µg/mL (*p* ≤ 0.001), confirming its strong cytotoxic potential. A concentration-dependent increase in cancer cell mortality was observed, particularly within the 25–200 µg/mL range, as confirmed by microscopic analysis. The incorporation of Ag, Cu, and ZrO_2_ nanoparticles into the biochar matrix enhanced its therapeutic efficacy, underscoring the potential of biochar-based nanocomposites as a promising strategy for neuroblastoma treatment. These findings suggest that biochar-derived nanomaterials could serve as an innovative platform for targeted cancer therapy, particularly for rare and treatment-resistant malignancies.

These three studies collectively demonstrate the diverse and innovative applications of biochar in cancer treatment. Whether through the modulation of cell signaling [139], enhancement of drug delivery systems [138], or targeting specific cancer types [136], biochar shows significant promise as a multifunctional material in anticancer therapy [135]. Given the promising preclinical results, further research focusing on optimizing biochar’s properties and evaluating its in vivo efficacy is warranted to fully realize its potential in clinical cancer treatment.

**Table 3:** Anticancer activities of modified biochar.

Source	Additional Material	Methods	Target	Effects	Ref.
Alder wood chips	Butyrate glycerides	Pyrolysis (450–700 °C, 3 h)	HCT116, HT29	- Making scaffolds using the adsorption capabilities of biochar - Enhanced inhibition of DNA replication and glycolysis due to biochar	[137]
Date seeds	Silver nanoparticles	Pyrolysis (550 °C, 10 °C/min)	HT29	- Reducing the toxicity of silver NPs through adsorption with biochar - Increased selectivity due to silver NP sustained release in biochar	[139]
Leaves of Pontederia crassipes L.	Zinc oxide nanoparticles	Pyrolysis (600 °C, 5 °C/min, 4 h)	MCF-7 human breast cancer cell	- Improves visible light absorption for photodegradation during complexation with biochar - 94.13% apoptosis of MCF-7 cells at 79.82 μg/mL ZnO/biochar NPs	[140]
Orange peels	Carbon nanostructures	Hydrothermal carbonization (240 °C, 600 rpm, 1 h)	A549	- Solving the low water solubility of traditional graphene-based materials with biochar - Nanobiochar caused cell death percentages to more than double with respect to the drug alone	[138]
Date seeds	Emericella dentata	Pyrolysis (550 °C, 2 h)	A549	- Inhibition of toxic Ag^+^ ion release through biochar binding to mitigate toxicity to tissues - BCL-2 and cyclin D1 downregulation in A549 with treatment of 50 μg/mL of biochar	[141]
Mangifera indica bark	Ag/Cu-ZrO_2_ nanostructure	Pyrolysis (650 °C, 35 °C/50 min, 3 h)	SH-SY5Y cell	- Using the adhesion ability of biochar to attach with metal oxides to form nanocomposites - 73.61% cell death was observed at a concentration of 200 µg/mL	[135]
Maize straw	Zinc oxide nanoparticles	Pyrolysis (600 °C, 6 h/vacuum-dried)	Streptomyces 85E strain	- Increase surface functional groups through biochar binding to improve adhesion and antimicrobial performance - Protein kinase inhibition to potential anticancer activity (1 mg/mL)	[136]

HCT116: human colorectal cancer; HT29: human colon adenocarcinoma; MCF-7: human breast cancer; A549: human non-small cell lung cancer; SH-SY5Y: human neuroblastoma.

### 3.3. Biochar in Biosensor Applications

**Figure 7:**
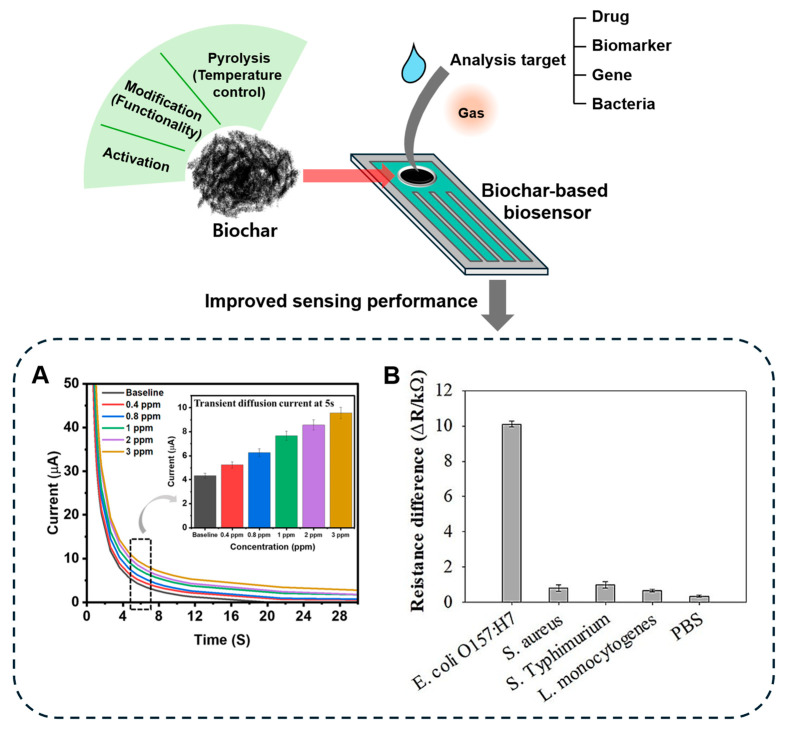
Summary illustration of biochar-based biosensors for detecting analysis targets involved human health. (**A**) Reprinted with permission from Ref. [155], Copyright © 2023. (**B**) Reprinted with permission from Ref. [156].

The application of biochar has been concentrated in the environmental field; however, recent research has explored its potential use in the medical sector, particularly as a biosensor [23] (Figure 7). Biosensor is a technology that converts biological signals into measurable electrical signals, enabling selective analysis of target substances [142]. Biochar offers several advantages for biosensor applications, including high adsorption capacity and surface area due to its porous structure, excellent ion exchange capacity, and cost-effectiveness [143,144]. The porous structure can be controlled according to modifications, which can contribute to improving sensor performance through target-specific optimization [145,146,147]. In addition, the abundant surface functional groups can be combined with various functional materials and bioreceptors such as enzymes or nucleic acids, making it suitable for use as a substrate for a biosensor [148,149,150]. The excellent ion exchange capacity is advantageous for detecting targets or detecting changes accordingly, which helps improve the sensitivity and efficiency of the sensor [151,152]. The fact that biochar is derived from organic waste has the economic advantage of reducing the cost incurred when manufacturing a sensor [153,154]. Various biosensors utilizing biochar have been developed for detecting drug residues and disease-related biomarkers, demonstrating their potential for point-of-care diagnostics and monitoring (Table 4).

The development of biosensors incorporating eco-friendly and cost-effective biochar contributes to the rapid and precise detection of drug residues in environmental and biological samples, aiding in drug abuse prevention and other biomedical applications. Gemeiner et al. designed an economical sensor using biochar derived from corn and wood, combined with ethylcellulose and screen-printing technology [157]. Using paracetamol as a model analyte, they demonstrated the feasibility of rapid and reliable quantitative analysis. Optimization of ethylcellulose concentration as a polymeric binder, pH conditions, and analytical methods was performed using biochar pyrolyzed at 470 °C. The biochar-modified carbon electrode exhibited more than a tenfold increase in sensitivity compared to the unmodified electrode, with a detection limit of 0.02 µM for paracetamol and a reliable detection range of 0.1 to 10 µM. The sensor was also tested with commercially available paracetamol and showed similar results compared to conventional methods, indicating its potential for use in drug analysis.

Additionally, various sensors are being developed to monitor sugar levels in the body for people with diabetes or for prevention. Among these, Kalinke et al. developed a biosensor by combining glucose oxidase and biochar [158]. Prussian blue nanoparticles were immobilized onto the surface of biochar via Fe ions, and glucose oxidase was covalently attached to the active residues of biochar. Prussian blue exists in a reduced state as Prussian white, and when glucose and enzymes react in solution, H_2_O_2_ is produced, which oxidizes Prussian white back to blue. The sensor using this cycle showed a linear detection range of 0.05–5.0 mM and a detection limit of 0.94 µM. Moreover, the repeatability and reproducibility of the sensor was confirmed through 10 repetitions, and the sensitivity to detect glucose without being affected by interfering factors such as ascorbic acid or lactic acid was confirmed. In this way, they detected glucose in real samples using human saliva and blood, showing recoveries of 84–104%, indicating that biochar can have a positive impact on biosensors for monitoring glucose in the body.

Biomarker tracking and monitoring represent key objectives in the sensor field, as they facilitate early detection of physiological abnormalities, disease diagnosis, and prognosis. Among various biomarkers, the level of ammonia in the body is associated with kidney disease and liver dysfunction. Banga et al. developed a wearable biosensor to detect and monitor ammonia emitted from the skin [155]. This biosensor utilized wood-derived biochar, hydrothermally carbonized under an N_2_ atmosphere following acid activation with H_3_PO_4_. Comparing three temperature conditions (400, 500, and 600 °C), the biochar treated at 400 °C was the most suitable in terms of capacitance and diffusion behavior because it was made of carbon material with less nitrogen content compared to the other conditions. The biochar-based biosensor had an ammonia detection limit of 0.4 ppm and measured with more than 95% accuracy down to 3 ppm. It was also highly sensitive to the target, showing more than three times the signal for ammonia compared to non-target substances such as nitrogen, carbon dioxide, and nitric oxide. As a result, they explained that it could be used as a point-of-care platform for kidney disease, with evidence that changes in ammonia concentration can be measured even when using a wearable device.

## 4. Challenges and Future Perspectives

Biochar has long been used in agriculture and forestry, and more recently, it has shown various potential applications in the biomedical field. Specifically, biochar is advantageous because it is low-cost, readily available, and produced through the environmentally friendly recycling of agricultural waste. However, in the biomedical field, particularly in the development of electrochemical sensors and biosensors, there are several key challenges to address. One of the main challenges in biochar-based biosensors is ensuring precision and repeatability. Biosensors require high precision in detecting target analytes. However, the variability of biochar, resulting from differences in feedstock types and pyrolysis conditions, can lead to inconsistent performance. This lack of standardization makes it difficult to ensure reliable sensor function. Additionally, biochar’s surface chemistry must be carefully controlled to improve selectivity and stability. In the absence of proper surface modifications, biochar-based sensors may experience non-specific binding, which can reduce their effectiveness. Another major limitation is biochar’s relatively low electrical conductivity compared to other carbon-based materials, such as graphene or carbon nanotubes. Since electrochemical biosensors depend on efficient electron transfer for signal generation, enhancing the conductivity of biochar is crucial for optimizing their performance. Furthermore, biochar’s susceptibility to biofouling may present challenges when used in biological samples. Proteins and other biomolecules can accumulate on the sensor surface, affecting accuracy and sensitivity. Ensuring the biocompatibility of biochar while preventing biofouling is crucial for its application in biomedical sensors.

To overcome these limitations, several advanced strategies have been proposed. Functionalizing biochar with nanomaterials such as metal nanoparticles (gold, silver, platinum) or conductive materials (graphene, carbon nanotubes) can significantly enhance its electrochemical properties, making it more suitable for biosensing applications. Additionally, chemical modifications, such as oxidation or amination, can introduce functional groups that enhance biochar’s selectivity for specific biomolecules. Optimizing pyrolysis conditions is another important approach. Adjusting parameters like temperature and activation methods can yield biochar with improved surface area, porosity, and conductivity, all of which are beneficial for biosensors. Furthermore, integrating biochar-based sensors with microfluidic technology or wearable devices can open new possibilities for real-time, on-site medical diagnostics. These innovations could make biochar-based sensors more practical for applications such as continuous health monitoring. Finally, advancements in computational modeling and machine learning can help predict and optimize biochar’s performance in biosensors. By analyzing the relationship between biochar’s structural properties and its electrochemical behavior, researchers can design more efficient and reliable sensors. Addressing these challenges and leveraging advanced technologies will enable biochar-based biosensors to become a sustainable, cost-effective alternative in biomedical applications.

Biochar-based materials hold significant promises in biomedical applications due to their high surface area, tunable porosity, and renewable, cost-effective sources. These characteristics enable efficient drug loading, making biochar a strong candidate for drug delivery, tissue regeneration, biosensing, and antimicrobial systems. Furthermore, biochar’s surface chemistry is highly adaptable, allowing for functional modifications that enhance specific biomedical properties. Preliminary in vitro studies have indicated low cytotoxicity and good biocompatibility, suggesting its potential for further development in vivo. However, biochar’s inherent heterogeneity, arising from variations in feedstock, pyrolysis conditions, and post-treatment methods, complicates standardization, which limits reproducibility and comparative evaluations. Additionally, there is a lack of comprehensive in vivo studies addressing long-term toxicity, biodistribution, biodegradability, and immunogenicity. Toxic impurities, such as polycyclic aromatic hydrocarbons (PAHs) and heavy metals, may also present risks if the pyrolysis process is poorly controlled. Biochar’s current exclusion from established regulatory frameworks poses further challenges to its clinical translation. Future research should focus on standardizing synthesis protocols, expanding in vivo studies, and integrating biochar with complementary materials to address these challenges and unlock new biomedical functionalities [1,26,163,164].

Additionally, biochar’s long-term stability and immunogenicity in biological environments pose challenges [23,165,166]. If biochar remains non-degradable in vivo, it may accumulate and cause undesirable immune responses or toxicity. Developing biodegradable biochar composites and understanding their degradation pathways can help mitigate these risks. Despite promising preclinical results, the translation of biochar-based drug delivery systems into in vivo applications and clinical settings faces several hurdles. Biological environments are complex, leading to unpredictable interactions that affect biochar’s stability and efficacy. Regulatory approval for new biomaterials requires extensive safety and efficacy evaluations, which can be time-consuming and costly. Recent studies have explored various aspects related to the clinical applicability of biochar-based drug delivery systems. These investigations propose strategies to enhance the in vivo potential of such systems by improving the biodegradability of biochar, reducing its immunogenicity, and enhancing its thermal stability. In particular, approaches involving the fabrication of composites with biodegradable polymers, the application of specific microbial consortia to promote biochar degradation, and the use of nanoparticle-based monitoring technologies have attracted increasing attention [167,168,169]. Nevertheless, further comprehensive investigations are required to elucidate long-term stability, immunogenicity, degradation pathways, immune responses, and regulatory challenges associated with biochar-based systems. To date, most research has remained at the basic research stage, and therefore, more refined and standardized follow-up studies are essential for its application in human patients.

Biochar presents several advantages for biomedical applications, including its low production cost, environmental sustainability, and customizable properties, especially when derived from agricultural or industrial biomass residues. However, converting biochar into biomedical-grade materials requires additional processing steps such as high-temperature activation, chemical purification, surface functionalization, and sterilization, which increase production costs. Large-scale production also necessitates consistent quality control, standardized synthesis protocols, and compliance with good manufacturing practices (GMPs), leading to higher operational and infrastructure costs. These factors can offset the initial economic benefits of biochar, making its economic feasibility dependent on regulatory compliance, safety validation, and processing requirements. Therefore, future research should incorporate life cycle assessments and techno-economic analyses to evaluate whether biochar can remain cost-effective while meeting the rigorous standards required for biomedical applications [170,171,172,173].

Reproducibility remains a key challenge for large-scale applications. Variability in raw materials, synthesis methods, and experimental conditions can significantly impact the consistency of results. Variations in feedstock composition, pyrolysis temperature, residence time, and local environmental factors can result in significant inconsistencies in the physicochemical properties and performance of biochar materials. Recent studies have highlighted that even minor deviations in production parameters can lead to substantial variability in biochar quality, limiting its functional predictability and scalability in industrial or environmental contexts . Critically, one of the most persistent issues in ensuring reproducibility is the lack of universally accepted standards for biochar classification and performance benchmarking. While attempts have been made to define basic characterization protocols, these are often insufficient to capture the full complexity and application-specific behavior of biochar. In many cases, results from different laboratories or production systems are not directly comparable due to differences in analytical techniques, reporting units, or data interpretation frameworks. To address these challenges, future research should focus on the establishment of standardized and scalable production protocols, robust characterization frameworks (e.g., surface area, porosity, functional groups), and inter-laboratory validation studies to ensure consistent outcomes.

Recent advancements in 3D printing technology have opened up new possibilities for the application of biochar in the biomedical field [174]. However, research on the nanoscale modification of biochar and its integration into 3D printing technology for biomedical applications is rarely found. Biochar can be incorporated into biocompatible scaffolds, enhancing tissue regeneration, wound healing, and drug delivery systems. By 3D printing biochar-based materials with precise control over their structure, innovative possibilities arise for creating customized implants, prosthetics, and drug delivery devices in an effective and biocompatible manner. Three-dimensionally printed biochar-based nanomaterials have a high capability to achieve significant improvements in tissue engineering due to their unique properties. These materials can create excellent structures for regulating cell behaviors such as signaling, differentiation, attachment, and migration. Additionally, their ability to interact with other biomolecules and respond effectively to light makes them valuable in a wide range of biomedical applications, including drug and gene delivery, cancer therapy, biological imaging, antimicrobial applications, and diagnostics. Despite the numerous advantages, several challenges remain in their clinical applications. For instance, the safety of biochar in the human body is a serious concern, and further studies are needed to confirm their in vivo applications. Nonetheless, the functionalization of biochar can enhance its compatibility and improve its long-term safety. In recent years, the application of bio-based minerals—such as biosilica and bio-calcium—in biomedical fields has gained considerable attention due to their unique properties, including biocompatibility, biodegradability, and the ability to promote cellular growth and tissue regeneration [175,176,177,178,179,180]. These bio-minerals, often derived from natural sources like plants and algae, have been extensively studied for their potential in drug delivery systems, wound healing, and bone regeneration [181,182,183].

In the future, functionalization technologies that enable precise control over the physicochemical properties of biochar are expected to advance significantly. In particular, biochar’s role is anticipated to expand in areas such as targeted therapy, precision medicine, and next-generation biosensors. Moreover, interdisciplinary research collaborations will be crucial in improving the stability and efficacy of biochar in vivo, thereby increasing its feasibility for biomedical applications. Through continuous research and technological innovation, biochar is poised to play a significant role in the advancement of future medical technologies.

## 5. Conclusions

This review explored the potential of functionalized and composite biochar materials for developing advanced biomedical applications. These materials demonstrate essential characteristics, including multifunctionality, enhanced bioavailability, and tunable surface properties, rendering them promising candidates for biomimetic scaffold development. Furthermore, recent advancements in 3D printing technologies have expanded the scope of biochar integration into biomedical innovations, offering new possibilities for tailored medical solutions.

In the context of antibiotics, biochar-based scaffolds and drug delivery platforms have been investigated to improve effectiveness in bone regeneration and targeted delivery, addressing various medical challenges. Moreover, the incorporation of biodegradable polymers has further enhanced biochar’s role in anticancer therapies and drug delivery systems. Functionalized biochar supports targeted drug delivery by increasing drug loading capacity and enabling controlled release, promoting higher precision in delivering anticancer agents to tumor sites while reducing overall systemic toxicity. Similarly, the integration of biochar into biosensor technology presents a promising approach for advancing biomedical sensing applications. Recent studies have demonstrated its potential in drug residue analysis, glucose monitoring, and biomarker detection, highlighting its versatility in point-of-care diagnostics and monitoring. Further research and optimization of biochar-based biosensors could enhance their sensitivity, stability, and practical applicability, contributing to the development of innovative and sustainable sensing technologies.

## Figures and Tables

**Table 1:** Advantages and disadvantages of different preparation methods. Each method offers unique benefits and limitations.

Methods	Advantages	Limitations	Ref.
Pyrolysis Carbonization	- High biochar yield with controllable properties - Controllable heating rate, temperature, and gas atmosphere - Produces valuable byproducts (bio-oil, syngas)	- High energy consumption - Long processing time	[46,58,59,60]
Hydrothermal Carbonization	- Suitable for wet biomass (no drying needed) - Functional hydrochar with oxygen-containing groups - Low-energy process	- Low graphitization degree limits conductivity - Long reaction process	[59,61,62]
Torrefaction	- Energy-efficient, low-temperature process - Produces biochar with moderate carbonization	- Limited adsorption capacity due to low carbon content	[63,64,65,66]
Slow Pyrolysis	- Maximizes biochar yield - Produces stable and durable biochar ideal for soil applications	- Energy-intensive process - Requires long residence time	[46,67,68,69,70,71,72]
Fast Pyrolysis	- Short processing time - Generates biochar with specific properties for energy storage or filtration	- Lower biochar yield compared to slow pyrolysis - Precise temperature control required	[71,73,74,75,76]
Laser-induced Carbonization	- Produces high-porosity biochar - Rapid process for creating structured biochar - Suitable for advanced applications	- Limited to small-scale or surface carbonization - Requires specialized equipment	[77,78]
Microwave-assisted Carbonization	- Quick and energy-efficient process - Uniform biochar production	- Difficult to control reaction temperature - Poor reproducibility for large-scale production	[79,80,81,82,83]
Ball Milling	- Enhances surface area and porosity - Useful for producing nanobiochar and biochar composites	- High energy input for extended milling - Generates fine powders requiring careful handling	[84,85,86,87,88]

**Table 4:** Biosensor applications of biochar for biomedical analysis.

**Source**	**Additional Material**	**Methods**	**Target**	**Effects**	**Ref.**
Corn and wood	Ethylcellulose	Pyrolysis (470 °C, 25 min) Screen printing	Paracetamol	- 20–280 times higher sensitivity due to biochar modification - LR of 0.1–10 µM and LOD of 20 nM in tablet sample	[157]
Coster cake	Prussian blue Glucose oxidase	Pyrolysis (400 °C, 5 °C/min for 60 min) Covalent enzyme immobilization	Glucose	- Prussian blue attachment and enzyme immobilization by biochar - Repeatability and reproducibility of more than 90% for 10 times - LR of 0.05–5 mM and LOD of 0.94 µM in human saliva and blood	[158]
Raw tea waste	Copper	H_3_PO_4_ treatment (60 °C, 30 min) Pyrolysis (500 °C, 1 h) Electrodeposition	Glucose	- Improved sensing performance by biochar modification - LR of 0.8 µM–1 mM and 1–5 mM - Nonenzymatic glucose detection with selectivity against interferences - 96% detection in fetal bovine serum	[159]
Wood	-	H_3_PO_4_ treatment Thermal carbonization (10 °C/min to 400 °C, hold for 3 h)	Ammonia	- Enhanced performance according to low nitrogen and high carbon in activated biochar - LOD of 0.4 ppm and higher sensitivity compared non-specific compounds - Device development and validation for wearable sensor	[155]
Mushroom	MoO_3_ ZnO	Thermal calcination (550 °C for 3 h, 2 °C/min)	Acetaminophen	- Faster electron transfer rate and electrochemical signal amplification due to biochar - LR of 2.5–2000 µM and LOD of 1.14 µM - High sensitivity in presence of interferences, blood sample, and medical tablet	[160]
Silk	Ni	Pyrolysis (800 °C) Electrochemical deposition	Glucose	- Improved catalytic efficiency through uniform Ni layer and active surface area due to biochar - LR of 1–1498 µM and LOD of 0.16 µM in 0.1 M NaOH with glucose - Noninvasive and nonenzymatic glucose detection in human saliva	[161]
Pine tree residues	MoS_2_ Gold nanoparticle	Pyrolysis (600 °C, 1 h) Hydrothermal method	Gene of *S*. *dysenteriae*	- PCR-free and DNA-based genosensing for pathogen detection - Improved electron transfer rate and electrode surface area by combination with biochar - LR of 0.01–10 pM and LOD of 9.14 fM - High selectivity compared to other bacteria sequences	[162]
Corn stalk	Anti-*E*. *coli* polyclonal antibody	Pyrolysis (250–300 °C) Steam activation (800 °C, 2 mL/min) Antibody immobilization	*E*. *coli* O157:H7	- Food pathogens detection using increased surface area of activated biochar and antibody immobilization - LR of 10^4^–10^7^ CFU/mL and LOD of 10^4^ CFU/mL without incubation	[156]
Sugarcane bagasse	SARS-CoV-2 S-protein receptor binding domain	Pyrolysis (5 °C/min, 700 °C) Dropping electrode modification	SARS-CoV-2 antibody	- Providing large surface area and functional groups of biochar for protein immobilization - LOD of 10 ng/mL in serum sample - 95% confidence, selectivity, and stability for 7 days based on 82.3% cutoff	[149]

*S*. *dysenteriae*: *Shigella dysenteriae*; *E*. *coli*: *Escherichia coli*; LOD: limit of detection; LR: linear range of detection.

## Data Availability

Not applicable.
